# Revisiting the recent European droughts from a long-term perspective

**DOI:** 10.1038/s41598-018-27464-4

**Published:** 2018-06-22

**Authors:** Martin Hanel, Oldřich Rakovec, Yannis Markonis, Petr Máca, Luis Samaniego, Jan Kyselý, Rohini Kumar

**Affiliations:** 1 Czech University of Life Sciences, Faculty of Environmental Sciences, Prague, 169 00 Czech Republic; 2 UFZ-Helmholtz Centre for Environmental Research, Leipzig, 04318 Germany; 3 Institute of Atmospheric Physics, Czech Academy of Sciences, Prague, 141 31 Czech Republic

## Abstract

Early 21st-century droughts in Europe have been broadly regarded as exceptionally severe, substantially affecting a wide range of socio-economic sectors. These extreme events were linked mainly to increases in temperature and record-breaking heatwaves that have been influencing Europe since 2000, in combination with a lack of precipitation during the summer months. Drought propagated through all respective compartments of the hydrological cycle, involving low runoff and prolonged soil moisture deficits. What if these recent droughts are not as extreme as previously thought? Using reconstructed droughts over the last 250 years, we show that although the 2003 and 2015 droughts may be regarded as the most extreme droughts driven by precipitation deficits during the vegetation period, their spatial extent and severity at a long-term European scale are less uncommon. This conclusion is evident in our concurrent investigation of three major drought types – meteorological (precipitation), agricultural (soil moisture) and hydrological (grid-scale runoff) droughts. Additionally, unprecedented drying trends for soil moisture and corresponding increases in the frequency of agricultural droughts are also observed, reflecting the recurring periods of high temperatures. Since intense and extended meteorological droughts may reemerge in the future, our study highlights concerns regarding the impacts of such extreme events when combined with persistent decrease in European soil moisture.

## Introduction

Since the beginning of the 21st century, Europe has experienced a series of extreme hot and dry summers (2003, 2010, 2013 and 2015)^1–3^. Over parts of Central Europe, mean summer temperature in 2003, was up to five standard deviations higher than the long-term mean^4^, and the 2015 summer was the hottest since 1950 across a large part of eastern and southwestern Europe^3^. In addition, recent non-summer periods have also been extreme, for example, air temperatures over Europe during the autumn of 2006 and winter of 2007 were ranked as the warmest in the last 500 years^5^. The implications of these extreme weather conditions were felt in the sectors of agriculture^6^, hydrology and water resources^7^, human health^8^ and ecosystem services^9^, among others.

During recent years, (palaeo-) climatic reconstructions of hydroclimatic variables have been introduced to describe streamflow^10,11^, floods^12^, average^13^ and extreme^14^ rainfall and drought characteristics^15^. Except for regional studies^16,17^, drought reconstructions often focus on characterization of meteorological droughts, i.e., a lack of precipitation, possibly combined with increased potential evapotranspiration. However, the impacts of hydrological drought (below-normal river discharge) are more heterogeneous in space and time than those of meteorological drought. This is due to significant links to hydrological preconditions, which are thus crucial for understanding and assessing the development of hydrological drought propagating from meteorological drought, as well as for assessing its impacts on water resources^2^. The same importance also applies to agricultural drought (soil moisture deficit) or groundwater drought (groundwater deficit) and its development from meteorological conditions^18^.

The majority of studies on recent hydrological droughts evaluate the drought properties in the context of records starting in the second half of the 20th century^1–3^. There are indications, though, that the main drivers of hydrological drought (precipitation and soil moisture deficits and high evapotranspiration^19^, with the latter linked to high temperature) had already reached recent levels in the more distant past. For instance, the highest daily temperatures in parts of Central Europe in 1540 were likely warmer than in 2003^20^. The spatial extents of the reconstructed meteorological droughts^15^ in 1616, 1893 and 1921 exceed or are at least comparable to those of the recent events. Further, documentary evidence indicates severe large-scale European droughts, e.g., in 1893^21^ and 1921^22^. By extending our time window into the past, we can thus more accurately assess the range of hydroclimatic variability^23^, and understand the extremity of recent drought events.

In the present paper, we use reconstructed climate fields of precipitation and temperature for the period 1766–2015 (provided by Casty *et al*.^24^) as input into a state-of-the-art hydrological model (mHM^25,26^) to quantify the extremity of recent drought events in a long-term European perspective. We provide concurrent investigation of droughts from meteorological (precipitation deficit), agricultural (soil moisture deficit), and hydrological (runoff deficit) perspectives. We contrast drought characteristics (i.e., severity, duration, and areal extent) of recent against historical large-scale droughts that were not available for assessments presented so far.

## Temporal variability of precipitation, soil moisture and grid-scale runoff

**Figure 1:**
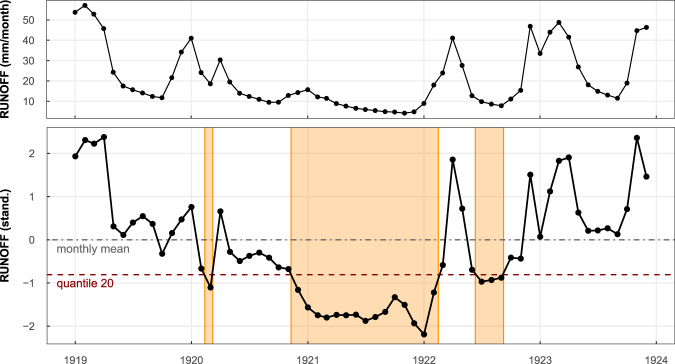
Estimation of Standardized Deficit Index (SDI) for runoff. Upper panel depicts the original time series of simulated runoff at a random grid cell in Central Europe for the period 1919–1923. For calculation of the SDI the values are first standardized by subtracting monthly mean and divided by standard deviation for each month separately. For the standardized series (lower panel) the values below zero (dot-dashed grey line) correspond to below-average runoff (e.g. the period from May 1920 to March 1922). Drought defining threshold (dashed red line) is calculated as the 20th percentile of the standardized series using quantile regression on time (to allow for non-stationarity, see Methods section). Drought event (orange area) starts when the series drops below the threshold and ends when it raises again. The SDI value is obtained as the cumulative sum of deviations from the threshold over the event for fluxes (precipitation, runoff), for system states (soil moisture) as the maximum deviation from the thresholds^27^ (since the state already integrates the fluxes). Figure was created in R (ver. 3.2.1, https://www.r-project.org/).

The mesoscale Hydrological Model (mHM^25^) simulates water fluxes and states on a 50 km × 50 km grid, which (under the set-up used for this study) covers most of Europe (excluding Scandinavia and the British Isles). The analysis presented here considers standardized time series (having zero mean and unit standard deviation in each month, see Fig. 1 and Methods section) of reconstructed precipitation^24^ and mHM simulated soil moisture and grid-scale runoff. The standardization is often used in regional/global drought studies^27^ to allow for comparison between regions exhibiting different hydroclimatic regimes. For example, the mean annual precipitation ranges from ca 300 mm in south Spain to more than 2000 mm in the Alps and thus, in absolute values, a relatively mild precipitation deficit from one location could correspond to severe drought at another location and vice versa. Note that by the term “grid-scale runoff” we mean the total water (surface, interflows and groundwater) that is produced at a grid scale before entering into the stream-network. In this way we can consistently compare between grid scale precipitation, soil moisture and runoff droughts, which would not be the case for the routed streamflow in rivers.

Drought is defined as a sustained and regionally-extensive period of below-average water availability^28^. In the long-term perspective, being it reconstructed series or climate change projection, it is not always clear whether a single (stationary) reference period can be considered for identification of below-average conditions since often the mean as well as other characteristics like variability of the distribution evolve in time. As a result the ecosystems and society slowly adapt to new hydroclimatic conditions (e.g. by transforming agricultural management) and what was considered drought may become normal in drier climate or conversely what was considered wet may become normal or drought in wetter climate^29^. Therefore in a context of long-term reconstruction of droughts, the changing temporal variability needs to be taken into account within drought definition.

**Figure 2:**
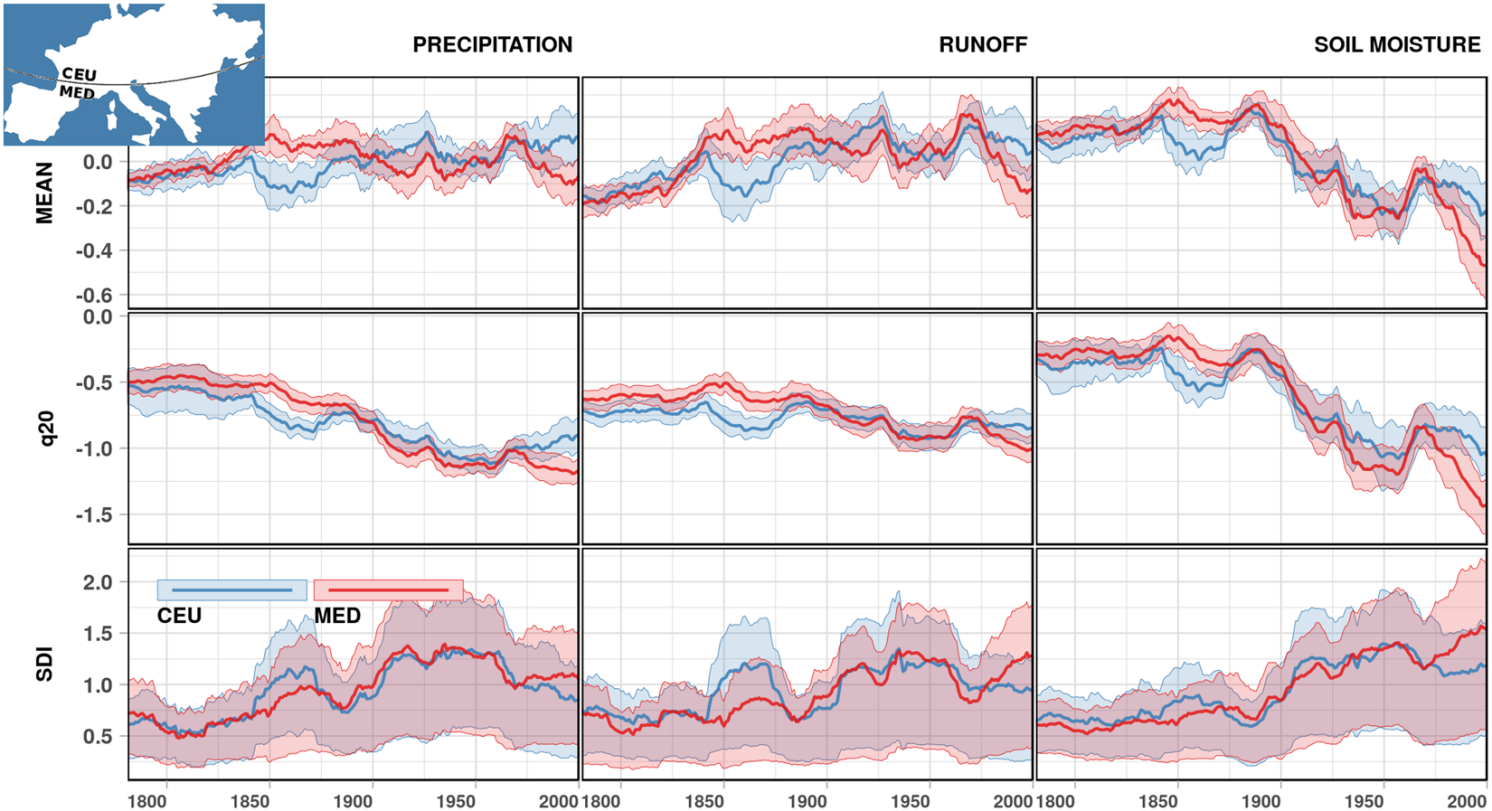
Temporal dynamics of 30-year moving average of the mean (top), 20^th^ percentile (q_20_; middle), and standardized deficit index (SDI; bottom) for the standardized precipitation (left), mHM-simulated grid-scale runoff (middle) and soil moisture (right). Values for the Central Europe (CEU) and Mediterranean (MED) regions are shown in blue and red colours, respectively. The thick lines correspond to areal means, and the envelopes span the range between the 5^th^ and 95^th^ percentiles of grid cell values for each region. The MED region is defined according to the IPCC Special Report on Extremes^19^: 30°N–45°N, 10°W–40°E, and the rest of the study domain corresponds to the CEU region. Figure was created in R (ver. 3.2.1, https://www.r-project.org/).

Figure 2 (top panels) shows the temporal evolution of 30-year moving average of the considered hydroclimatic variables averaged over the Mediterranean (MED) and Central European (CEU) regions. These regions are based on the IPCC Special Report on Extremes^19^. The precipitation over a relatively long time span of 250 years (1766–2015) slightly increases over the CEU and decreases over the MED regions. There has been, however, a pronounced reduction in precipitation in the MED region since 1970, which is consistent with the findings of previous observation-based studies^19,30^. The dynamics of grid-scale runoff closely follows that of precipitation, while a substantial drying trend is observed in soil moisture across both regions since the beginning of the 20th century.

Regardless of the increase in mean precipitation and grid-scale runoff in CEU, the lower quantiles of the distribution, which are more relevant to drought, are decreasing for all three variables in both regions. This is illustrated in Fig. 2 (middle panels) for 30-year moving average of the 20th percentile (q_20_) of the distribution of standardized variables. The strongest decreasing trend is noticed in soil moisture, followed by precipitation and runoff, across both the CEU and MED regions (see Supplementary Section 3.1 for more detailed assessment of trends).

Drought severity is frequently expressed using a threshold level approach through deficit index^27,29,31^. In this approach a drought event starts when the value drops below selected threshold (often q_20_) and ends when it rises again. Deficit index for an event (sometimes called deficit volume) is defined as a cumulative deviation from the threshold for fluxes (precipitation and grid-scale runoff) or a maximum deviation for states (soil moisture). The deficit index needs to be standardized^32^ to allow comparison across different climates (as explained above). In addition, due to non-stationary climate, the threshold defining droughts may vary in time^29,33^ as also discussed above. The time-varying threshold (quantile regression based q_20_) is therefore used here for calculation of the standardized deficit and the value is further referred to the Standardized Deficit Index (SDI; see Methods for more details) expressing drought severity. Note, that unlike popular Standardized Precipitation Index and similar indices, the SDI (or deficit index in general) does not involve any distribution fitting. In addition, since SDI does not relate to a specific variable we explicitly distinguish SDI for precipitation, SDI for soil moisture and SDI for grid-scale runoff throughout the paper.

The SDI exhibits substantial heterogeneity across both the CEU and the MED regions (Fig. 2; bottom panels) compared to the average and q_20_, which signifies the degree of non-linearity in the drought-generating processes. In general, we find a relatively strong increase in SDI for all variables in the first half of the 20th century. After 1950, this increase continues for precipitation in the western part of MED and CEU, for soil moisture in MED and western CEU and only partly for grid-scale runoff in MED (Supplementary Fig. S7, Supplementary Table S1).

## Spatio-temporal variability of drought over the last 250 years

**Figure 3:**
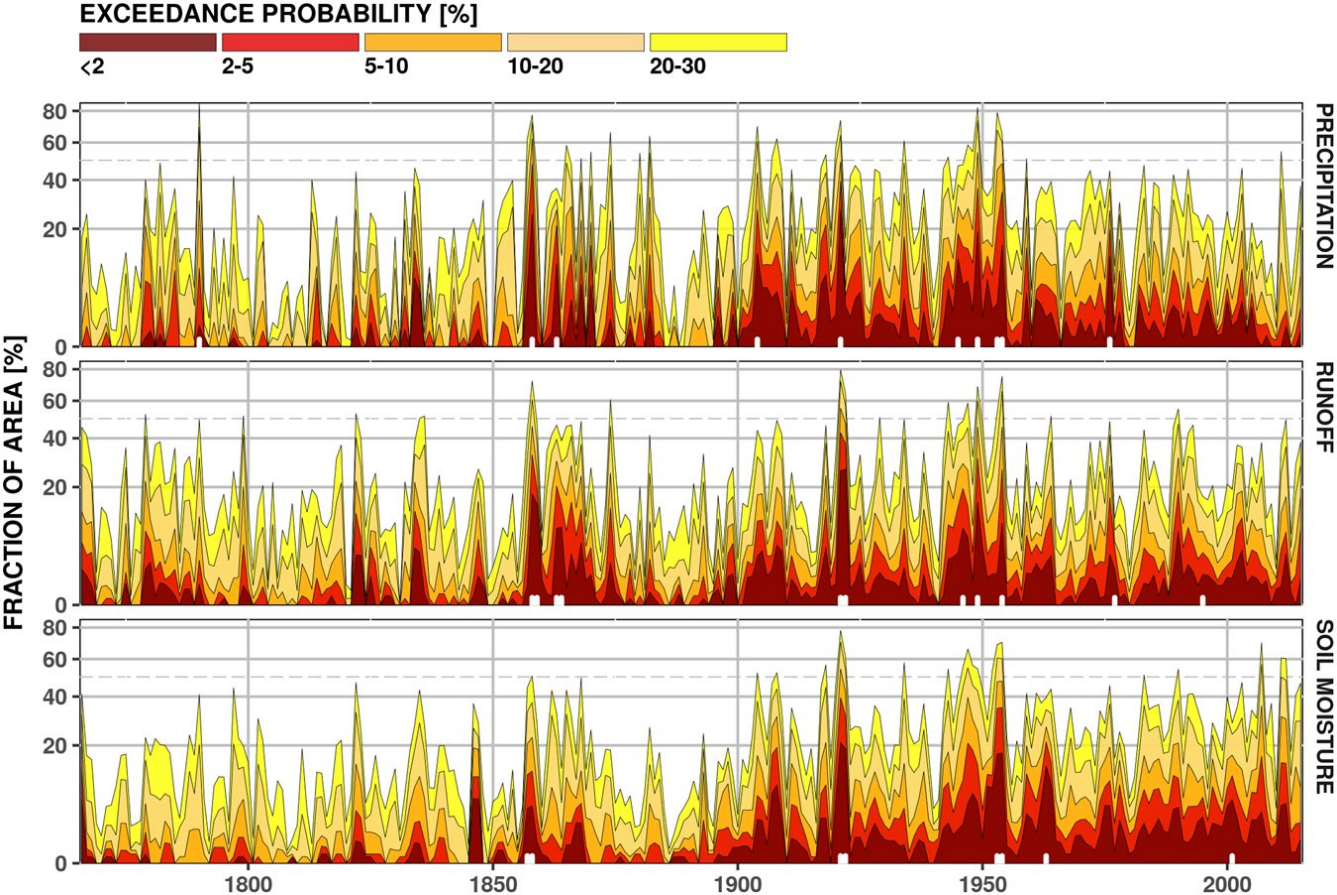
Time series of area under drought over the whole study domain for precipitation (top), grid-scale runoff (middle) and soil moisture (bottom). Colours correspond to SDI exceedance probability - the lower the exceedance probability the more severe the drought. White ticks indicate extreme drought events with respect to drought severity and areal extent as identified in Fig. 4. Figure was created in R (ver. 3.2.1, https://www.r-project.org/).

Figure 3 shows the temporal dynamics of drought areal extent for different exceedance probability levels of drought severity (see Supplementary Section 1.4). The areal extent is estimated as percentage of grid cells relative to the entire study domain. The decade of 1945–1955 can be classified as a period with the most extreme and long-lasting droughts during the last 250 years that appear in all three compartments of the water cycle. In general, our reconstruction of hydrological and soil moisture droughts agrees well with the past documented large-scale drought events that occurred in 1858–59, 1921–22, and 1949–50, and covered at least 20% of the study domain. The years 1921 and 1949–50 are found to have spatially extensive dry summers according to the Palmer Drought Severity Index (PDSI)^34^; with the severity of the 1921 drought was such that it led to some of the very first studies on drought^22^. The periods 1857–1858, 1921–22 and 1953–1954 can also be found in palaeoclimatic reconstructions of European drought^15^ and regional reconstructions of Rhine streamflow^35^ and Bavarian precipitation^36^. The literature is not as extensive for the Mediterranean, but there is strong evidence for the 1945 and 1949 droughts over the Iberian Peninsula^37^. The period of 1942–1953 is regarded as one of the driest across the whole MED region^38^.

Ample differences in the areal extent of drought are also found depending on the variables used for identification of drought. The meteorological droughts exhibit spiky behaviour, inherently related to the erratic temporal variability of precipitation, whereas the soil moisture droughts show more persistent behaviour (Fig. 3). The variability of the areal extent of hydrological (grid-scale runoff) drought results from the combination of fast and slow flow components, which signify the modulating effects of the terrestrial land (sub)-surface properties in the propagation of meteorological droughts to runoff and soil moisture^18,31^. For example, the meteorological drought in 1904 does not propagate into grid-scale runoff and soil moisture directly but with a considerable time lag and attenuation. Similarly, the hydrological and soil moisture droughts during 1859 and 1922 are largely affected by the extreme meteorological droughts occurring in the preceding years of 1858 and 1921, respectively.

Based on the temporal evolution of drought areal extent during 1950–2015, we find a significant increasing trend for soil moisture droughts and a decreasing trend for meteorological droughts for a number of SDI exceedance probability classes (Fig. 3). This result highlights the enhancing role of temperature increase on soil moisture droughts in recent decades, since it has been demonstrated that the temporal variability of soil moisture is mainly driven by climate. When changes in vegetation are small compared to the climatic fluctuations, i.e. during the last 30 years, vegetation processes can be considered more as a “regulator” for drying or wetting the soil^39^. More insight regarding the effect of temperature on soil moisture droughts can be sought in the well-studied 2012–2014 California drought, which has been regarded similar to the European 2003 drought in terms of concurrent low precipitation and extreme temperature^40^. What is remarkable with the California drought is that although it is found to be the most severe drought of the last millennium in terms of cumulative severity, the corresponding precipitation deficit is not unprecedented^41^. It is important to note that the latter is identified as the main driver of this multi-year drought, due to a persistent atmospheric circulation pattern that blocked oceanic moisture transport^42^. Therefore, the record high temperatures are considered to have exacerbated the dryness^43^, even though the onset and duration of the drought are also controlled by circulation-related precipitation deficit^44^.

## Extreme drought events

**Figure 4:**
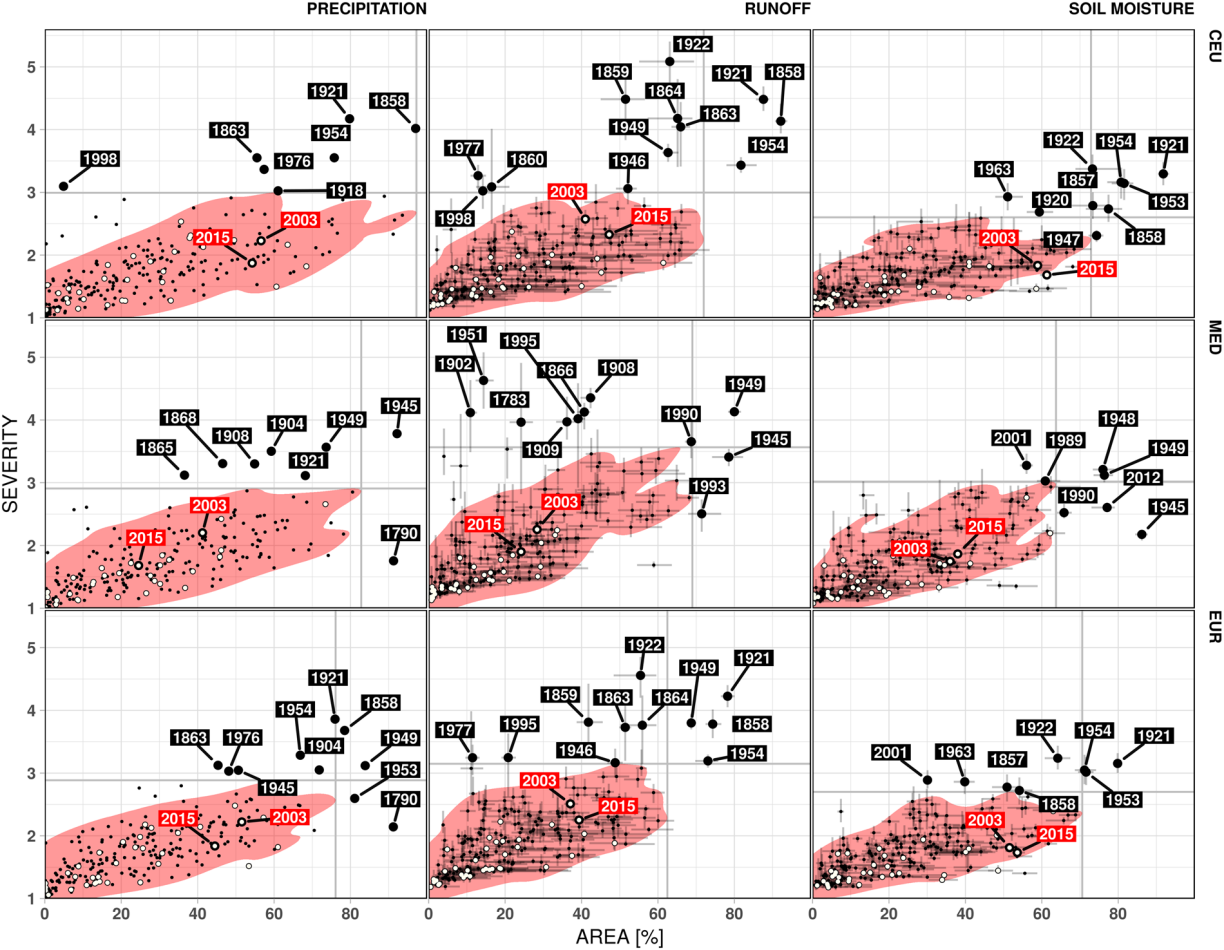
Relation between the area under drought and drought severity for precipitation (left), grid-scale runoff (middle) and soil moisture (right) over the Central Europe (top) and Mediterranean (middle) regions and all of the study domain (bottom), based on annual values. The red areas contain 95% of all drought events. The points correspond to the ensemble medians, and the lines span the interquartile range. Values for 2003 and 2015 are labelled in red. White points indicate droughts initiated in the vegetation period. Vertical (horizontal) thick grey line indicates maximum areal extent (severity) within the 95% envelope (red area). Error bars represent the composite uncertainty for the soil moisture and grid-scale runoff drought characteristics due to temporal disaggregation of meteorological forcings and model parameterization. Figure was created in R (ver. 3.2.1, https://www.r-project.org/).

To further identify the most extreme drought events over the past 250 years, Fig. 4 depicts the bivariate relationship between annual areal drought extent and corresponding severity computed over grid cells with SDI values greater than one. The extreme drought events are identified as those events falling outside the 95% quantile envelope of the bivariate distribution (see Methods Section for further details). Overall we find the droughts occurred in 1858–59, 1921–22 and 1953–54 as the most extreme events. In all three regions (CEU, MED, and entire EU), the extremely low precipitation falls outside the 95% quantile envelope only during the first year of the drought, while the runoff and soil moisture droughts persist to the next year. This distinguishing time lag and the attenuation of drought events in propagation of precipitation deficits to grid-scale runoff and soil moisture droughts suggest that it usually takes 1–2 years for runoff and soil moisture drought to recover from the precipitation deficit in such large-scale events.

Notably, these drought events affect the Mediterranean rather marginally, which suggests a different drought regime in the Mediterranean than the rest of Europe. In southern Europe, the most pronounced events include the droughts of 1945 and 1949, while there are also a number of other severe, mainly runoff, droughts with more confined areal extents (probably due to the characteristic spatial heterogeneity of the region). An exception is the drought of 1949, which is also evident in the CEU runoff, but this seems to be the only synchronous event between the two regions. Finally, the sharp decrease in soil moisture during the last century is also reflected in the increased frequency of soil moisture droughts; the most extreme events beyond the two major droughts (1945 and 1949) emerge in the last 30 years, namely, 1989, 1990, 2001 and 2012.

To this end, the monthly propagation of drought in the three most extreme cases in CEU is examined. These cases can all be classified as wet-to-dry season droughts^28^, i.e., their precipitation SDI starts its monotonic increase during the late summer/early autumn of the previous year, with the soil moisture decrease occurring in autumn and the response in runoff during winter. Interestingly, excess PET remains rather low in all three cases. On the other hand, the initiation of the 21st-century droughts can be found in mid-spring to mid-summer of the same year and is synchronous with a strong increase in PET. This result is in good agreement with a recent study on the hydroclimatic changes across Central Europe^45^, which claims that the onset of recent droughts in this region is more likely to be related to the temperature increase than to any major decrease in precipitation.

It becomes apparent that the recent 2003 and 2015 droughts are not such extreme events from a continental perspective. Our findings suggest that they exhibit considerably lower severity and areal extent with respect to the multi-year 1858–59, 1921–22 and 1953–54 droughts in all of the investigated variables. In fact, for runoff (soil moisture) there are 49 (110) past drought events with larger severity and 50 (25) events with larger area compared to recent events. Similar to the California case, the recent droughts are linked to low precipitation caused by blocking atmospheric circulation patterns combined with record-breaking temperature conditions^1,3^. However, when examined from a 250-year perspective, the precipitation deficit over the entire study domain is found to be not large - according to mean annual precipitation deficit volume, it ranked 23rd and 73rd for 2003 and 2015, respectively. In both years, the decrease in precipitation occurred solely during spring and summer. In 2003, precipitation returned to normal conditions during the succeeding autumn, while the severity of the 2015 drought was possibly limited due to the wet preceding winter over large part of Europe^2,7^. Therefore, the annual statistical properties of the droughts considered here might mask their extremity occurring at finer time scales and with respect to different drought-generating processes. Indeed, when only droughts initiated during the vegetation period are considered, the 2003 and 2015 events are the most intense events in the 250-year of reconstructed dataset with respect to severity and spatial extent of runoff drought (see white dots in Fig. 4). This observation reveals that recent seasonal droughts are generated by different physical processes compared to their historical long-term counterparts.

Runoff droughts in 2003 and 2015 are especially pronounced in CEU, but they count among the most severe vegetation-period droughts also in the MED region. Similarly, the spatial extent of the area affected by agricultural drought is considerable (60% of the CEU area and 35% of the MED area) and rather atypical for this drought type, with more severe and extended droughts only in 1917 and 2011 in MED (Fig. 4). Figure 4 also shows the error bars representing the composite uncertainty for the modelled soil moisture and grid-scale runoff drought characteristics due to temporal disaggregation of meteorological forcings and model parameterization (see Methods Section for more details). The majority of the events outside the 95% envelope, and especially the most extreme ones, are clearly distinguishable regardless of the uncertainty. The uncertainty was further decomposed into parametric and forcing parts. For runoff drought area (severity) the parametric uncertainty is on average 3.5 (3.6) times higher for runoff and 2.2 (2.3) for soil moisture than the forcing uncertainty. In absolute values, the mean of the drought area (severity) due to the parametric uncertainty ranges between 22 ± 8.5% (±0.28) for runoff and ±3.9% (±0.12) for soil moisture drought area (severity). These findings suggest that the disaggregation effect in the reconstructed climate input is negligible and that also the model parameterization introduces only a small uncertainty in the modelled results.

**Figure 5:**
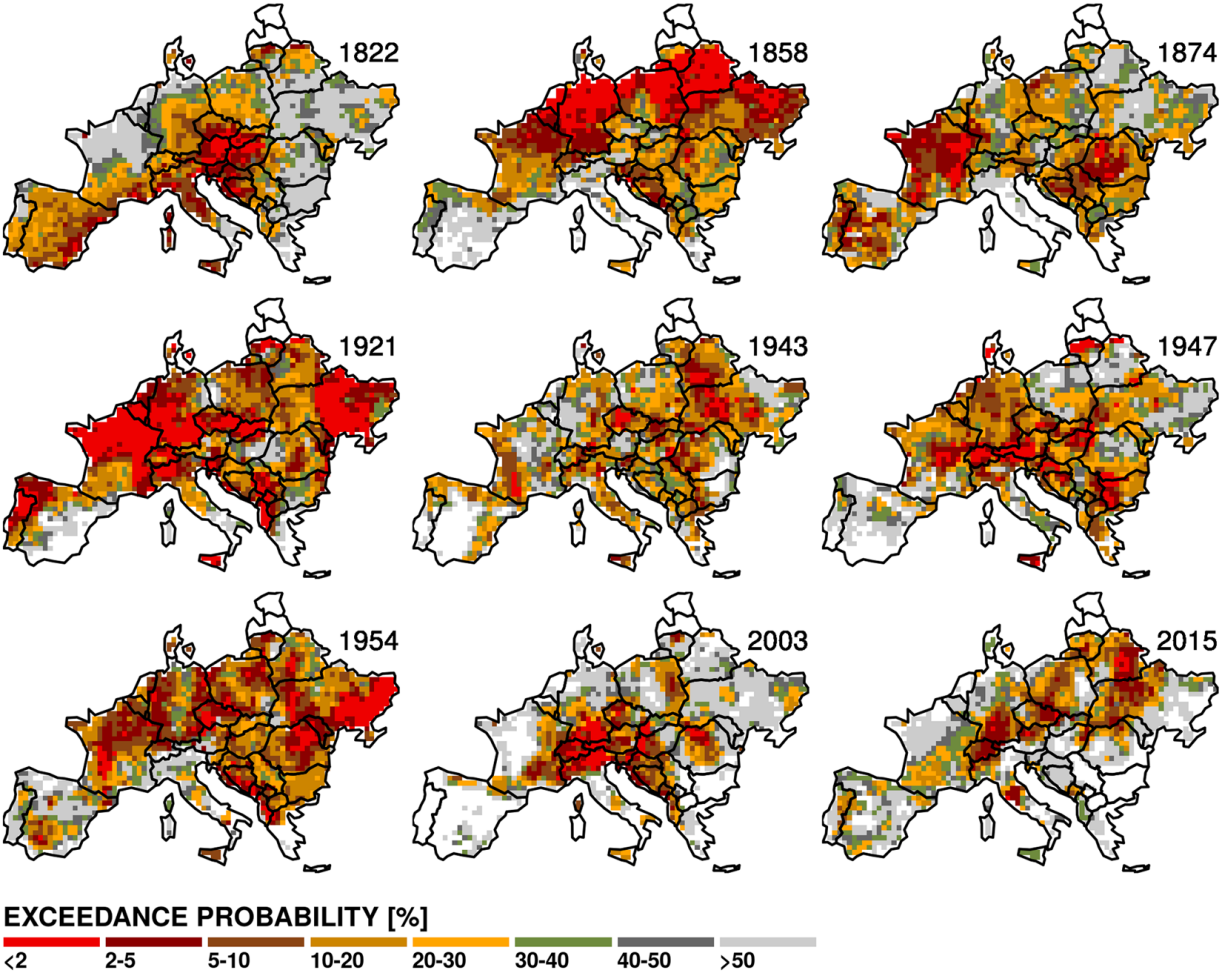
Exceedance probability of large-scale extreme grid-scale runoff drought events identified in Fig. 4 together with those for 2003 and 2015. Note that low exceedance probability corresponds to large drought severity. Figure was created in R (ver. 3.2.1, https://www.r-project.org/).

Similarly to the discussion of the temporal domain and drought type, the fact that the recent drought events from the continental perspective are not as extreme as previously perceived can be misleading regarding their severity or rarity at finer spatial scales. Therefore, a comparison of selected widespread extreme drought events with the 2003 and 2015 droughts is performed (Fig. 5, see also Supplementary Figs S9 and S10). Although locally (e.g., in southern France and Germany, Switzerland, Austria and the Czech Republic) the SDI for grid-scale runoff in 2003 and 2015 reaches extreme levels (i.e., the exceedance probability is low), the droughts of 1858, 1921 and the multi-year drought series around 1950 appear to be the more intense, covering a large part of Europe and reaching high severity levels. This difference in severity becomes even more apparent if the SDI annual values per grid cell are examined for each drought event. For instance, during 1921, 50% of the grid cells present runoff SDI twice as large as average (i.e., SDI > 2), and 20% more than four times the average (SDI > 4; mainly in France, Germany and eastern Ukraine). In 2003 and 2015, there are less than 10% of grid cells with SDI larger than 2 and almost no grid cells with SDI > 4, respectively (see Supplementary Fig. S11). While similar differences are found in the distribution of precipitation SDI (the area with high SDI values is considerably larger in 1921 than in 2003 or 2015), the distribution of soil moisture SDI for recent droughts approaches that of 1921 more closely. This raises concerns about the consequences of extreme meteorological droughts in combination with soil moisture deficits enhanced by a warmer climate.

Finally, the spatial patterns of our simulations are also in good agreement with most of the major drought events presented in the international database of text-based reports^46^. These events include the soil moisture droughts in the western Mediterranean and Central Europe during 2004–2008 and 2011–12, the severe 1989–1990 meteorological/hydrological drought over the Mediterranean and southern France and the 1975–1976 drought covering France, Germany and Eastern Europe. The exact geographical characteristics of these events can be seen in detail at https://shiny.fzp.czu.cz/KVHEM/drought/. In most cases, the patterns of precipitation and soil moisture droughts are markedly different from those of runoff, highlighting the importance of different drought drivers and drought propagation. It must be noted, however, that in recent events runoff droughts are at particular locations more severe (have lower SDI exceedance probabilities) than meteorological/soil moisture droughts, although the latter have broader spatial extent (compare Fig. 5 to Supplementary Figs S9 and S10). In addition, the droughts over the Mediterranean seldom affect the whole area (an exception is the extensive drought event of 1945), following the east-west climate dipole that has been detected as the dominant component of hydroclimatic variability in the Mediterranean^47^.

Although the recent European droughts are found to be the most extreme droughts initiated in the vegetation period (in the 250-year reconstructed dataset), they are much milder and more limited in both space and time with respect to their large-scale predecessors. For instance, compared to 2003 and 2015 events, the area affected by the most extreme hydrological droughts and drought severity was larger by 40% and 55%, respectively. The severity is larger even by 70% for the soil moisture drought. The reason that the recent droughts were not developed to a similar extent or severity lies in the wet preconditions and/or the rapid increase in precipitation that terminated them^2^. This interpretation could also be in good correspondence with the positive trend in precipitation observed in mid to high latitudes over Europe since the beginning of the previous century^48^; and with the study of Kwon *et al*.^49^ showing that a multi-decadal wetting trend can make recent droughts appear quite extreme, while such droughts could have been common in the previous centuries. Nevertheless, the California drought has already taught us that in changing hydroclimatic conditions, the prolonged periods of precipitation deficits, even when not reaching the most extreme levels, can lead to large-scale severe droughts. Further analysis of drought impacts under conditions such as large precipitation deficit and/or higher temperature, remains a challenge for future research, and the combination of climate reconstructions with an ensemble of hydrological models could help us to understand and describe what might occur in a warmer climate.

Our study relies on the reconstructed climate fields of precipitation and temperature. In the reconstruction study^50^ the estimated uncertainty due to reconstruction is highest in winter (up to 20 mm/season) and lowest in summer (up to 7 mm/season). Validation of gridded data against point observation is always challenging due to scale mismatch. However, we performed a validation of modelled river flows and partly also groundwater levels against available observations (see Supplementary Material for more details). Especially at the standardized scales - relevant to droughts - the mHM simulated runoff and groundwater levels correspond quite well to observed values. In addition, this also holds for the SDI estimates calculated from simulated and observed runoff time series.

This study has primarily evaluated the impact of climate conditions on drought development. Therefore, other aspects such as time-varying model parameterization (e.g. due to vegetation changes) were not fully addressed, mainly due to lack of reliable available data. Nevertheless, some recent studies^51,52^ suggest that the effects of land use changes on runoff are much less important compared to that of the climate variability; e.g. Li *et al*.^53^ reports this difference to be close to one order of magnitude for large catchments.

Additionally, the hydrological drought is often considerably modulated by groundwater. In the present study we did not tackle the surface-groundwater interactions in depth, partly due to simplification of groundwater dynamics within mHM and relatively coarse spatial resolution (see Methods Section for more detail). The independent verification of simulated groundwater anomalies, however, revealed good correspondence with observations (Supplementary Fig. S4) and therefore we do not expect that groundwater representation in mHM would lead to considerable bias in hydrological drought characteristics. More detailed assessment of groundwater and vegetation controls on drought remain challenging topics for future research.

## Methods

### Reconstructed climate fields

The assessment of drought characteristics during the last 250 years is performed across a large part of Europe (excluding Scandinavia and the British Isles) at a spatial resolution of 0.5° × 0.5° and a monthly time step. We employ gridded fields of land surface precipitation and air temperature for the period 1766–1900 (already available from Casty *et al*.^24^), which were reconstructed by up-scaling the available station data using principal component regression^24,50^ on the CRU TS dataset^54^. In line with previous studies^24,50^ the CRU TS dataset^54^ was used for the period 1901–2015. We derived the monthly estimates of potential evapotranspiration considering monthly mean temperature and the approximations for extraterrestrial solar radiation^55^. We refer to this dataset (monthly time series of land surface precipitation, air temperature and potential evapotranspiration for the period 1766–2015 on a 0.5° × 0.5° grid) hereafter as the reconstructed climate fields/data.

### Model setup and experiment design

We use the grid-based, spatially explicit mesoscale Hydrological Model (mHM)^25,26^ to reconstruct monthly fields of grid-scaled runoff and root-zone soil moisture over the European domain since 1766. The numerical approximations and conceptualizations employed in the mHM are similar to well-known hydrological models such as the HBV^56^ or VIC models^57^, accounting for canopy interception, snow accumulation and melting, soil moisture dynamics, infiltration and surface runoff, evapotranspiration, subsurface storage and discharge generation, deep percolation and base flow, and flood routing. A non-linear separation scheme based on the HBV model^56^ is implemented to partition incoming net rainfall into soil moisture and in/ex-filtration in root-zone soil layers. The evapotranspiration from different soil layers is modelled based on available soil moisture stress and the fraction of vegetation roots in each soil layer. mHM considers fast and slow flow components for the grid-scale total runoff production. The fast flow component is represented through a combination of a threshold based quick interflow part and a relatively (slower) quasi-permanent interflow part with different recession constants. The slow flow component in mHM, which reflects the groundwater contribution to runoff, is modelled as an outflow of a linear reservoir with varying recession constants depending on the spatial heterogeneity of the underlying aquifer properties. The total runoff generated at every grid cell is routed to its neighbouring downstream cell using the Muskingum routing algorithm. The model uses the novel multiscale parameter regionalization scheme to account for sub-grid variability of landscape attributes and model parameters that allows the seamless prediction of water fluxes and states across a range of spatial scales and locations^25,26,58^. To date, the mHM has been previously in depth parameterized and successfully evaluated against multiple datasets (including evaporation, changes in the terrestrial water storage anomaly, soil moisture) at multiple spatial resolutions and over a large number of river basins world-wide^25,26,58–64^. In addition, a multi-model investigation conducted recently showed a better skill of mHM in capturing the dynamics of river flow across Europe compared to more complex models like Noah-MP and PCRGLOB-WB^65,66^. Detailed model evaluation is further presented in Supplementary Section 1 and corresponding Figs S1–S4. More details on model conceptualisation and applications of mHM may be found at http://www.ufz.de/mhm.

To enable the mHM runs at a daily time scale, we disaggregate the monthly reconstructed climate fields using a non-parametric k-Nearest Neighbour resampling approach^67,68^; i.e., the monthly values (sums of precipitation or averages of temperature) are distributed over the individual days in the same way as in the analogue month. The analogues for monthly reconstructed climate fields are searched in the E-OBS^69^ dataset (v14.0, 1950–2016), which is aggregated to a monthly time scale. The similarity between the aggregated E-OBS and reconstructed months is quantified considering spatial correlation of monthly total precipitation and mean air temperature. For each month of the climate reconstruction, the analogue E-OBS month is randomly selected from the most similar months with probability of selection proportional to the similarity between months. The procedure can be summarised formally as follows:

1. Aggregate E-OBS precipitation and temperature from daily to monthly time scale and denote this *Em*[*y*, *m*], with *y* the year from 1950 to 2016 and *m* = 1, …, 12 the month.
2. Select reconstructed precipitation and air temperature for month *m* in year *y* and denote this *Rm*[*y*, *m*].
3. To evaluate the similarity between *Rm*[*y*, *m*] and E-OBS records, calculate average spatial correlation between *Rm*[*y*, *m*] and *Em*[*Y*, *M*], with *Y* = 1950, …, 2016 and *M* = *m* − 1, *m*, *m* + 1, i.e., the analogues can be selected from any E-OBS year but have to come from the closest months to *m*. Since we have 67 years of E-OBS data, we have 3 × 67 = 201 candidates for disaggregation.
4. Rank the individual year-month combinations of *Em*[*Y*, *M*] according to the mean correlation, with the rank *k* = 1 for the largest correlation (i.e., the nearest neighbour).
5. Select 15 nearest neighbours of *Rm*[*y*, *m*] from *Em*[*Y*, *M*], i.e., those year-months with the largest correlation (*k* = 1, …, 15)
6. From the 15 nearest neighbours sample one neighbour with probability of selecting neighbour *k* equal to
  1 $$\frac{1}{k}\frac{1}{{\sum }_{i=1}^{15}1/i}$$
  denote *y*_*s*_ and *m*_*s*_ the year and month corresponding to the sampled neighbour.
7. Distribute monthly precipitation total and monthly air temperature of *Rm*[*y*, *m*] into days in the same way as observed for *Em*[*y*_*s*_, *m*_*s*_].
8. Repeat the steps 2–7 for each year and month of the reconstructed climate data.
9. Repeat the whole procedure several times if needed.

Using this approach, we construct a 10-member ensemble of daily climate forcings for 1766–2015. In addition, 10 good sets of mHM parameters^62^ are used for hydrologic simulations for every forcing set, which yields 100 ensemble simulations in total.

Note that the procedure described above provides daily climate fields which are, after aggregation to monthly time scale, identical to the original reconstructed climate fields. In other words, the sampling introduces only the within-month variability and retains the monthly variability from the original (reconstructed) data. Thus the possible errors/biases introduced by disaggregation will influence our analysis (which is done on monthly scale) only to the extent to which monthly grid-scale runoff and soil moisture are influenced by distribution of monthly precipitation and air temperature into individual days. It has been shown that within-month variability of precipitation and temperature influences to some extent distribution of runoff at monthly time scale^70^. For instance in our ensemble, the variation of monthly runoff due to within-month variability is on average around 3%, while for soil moisture this variation is less than 1%, which is acceptable. The uncertainty of drought severity and area under drought due to within-month variability is discussed for the most extreme drought events together with Fig. 4 and in general does not influence substantially our conclusions.

### Standardized deficit index (SDI)

To analyse the drought characteristics, a deficit index, which measures a cumulative deviation below a pre-selected threshold (for fluxes, i.e. precipitation and grid-scale runoff) or maximum deviation below this threshold (for states, i.e. soil moisture)^27,71^, is considered. The cumulation of the deviations below a threshold transforms the flux (runoff) into state (volume of lacking water or its dimensionless indicator in the case of standardized data) which can be then easily compared to soil moisture deficit. The runoff/soil moisture data for each grid cell are standardized prior to the calculation of the deficit index by subtracting the mean and dividing by the standard deviation for each calendar month separately. Standardization facilitates the comparison across space and time, prevents large differences between climate types^32^ and removes seasonality. A low percentile of runoff (soil moisture) is usually considered as a threshold defining drought. To account for the changes in the distribution of runoff (soil moisture, precipitation) over the period 1766–2015 (see the Temporal variability of precipitation, soil moisture and grid-scale runoff section in the main text), the 20th percentile estimated by a linear quantile regression^72^ (conditional on time) is taken as a threshold for defining drought events at each grid cell. This value is in line with the definition of drought as the deviation from (temporarily evolving) normal conditions^31^, considers adaptation to changing conditions^73^ and has been used in previous studies^29,33^.

We analyse time series of the maximum annual deficit index scaled by the average annual maximum deficit index at a grid cell (denoted hereafter as the Standardized Deficit Index; SDI). The value of SDI is sometimes referred to as drought severity throughout the paper. The SDI is estimated separately for each grid cell and variable under consideration (i.e., precipitation, runoff, soil moisture).

### Identification of extreme drought events

At each 0.5° grid cell, the drought events identified over the last 250 years are ranked from the highest to lowest severity, and the exceedance probability (*p*) is calculated as *p* = (*r* − 0.3)/(*N* + 0.4)^74^, with *r* the rank and *N* the total number of drought events for a given grid cell. For each year, the area under drought is calculated for different exceedance probability classes following the US Drought Monitor (http://droughtmonitor.unl.edu).

To identify the most extreme events, we calculate for each event the area with SDI > 1, i.e., with SDI larger than average. Using a two-dimensional kernel density estimation, we derive the envelope covering 95% of the observed drought events with respect to average drought severity and areal extent for each variable (precipitation, runoff, soil moisture) and region (Central Europe – CEU, Mediterranean – MED and the whole study domain – EUR). Furthermore, for this analysis, we exclude events with smaller severity or areal extent than any event inside the 95% envelope. The remainder represents the extreme drought events.

### Data availability

Reconstructed precipitation and temperature, which were used to drive the mHM model are available at ftp://ftp.ncdc.noaa.gov/pub/data/paleo/historical/europe/casty2007/. The HadCRU TS product is available through http://catalogue.ceda.ac.uk/uuid/edf8febfdaad48abb2cbaf7d7e846a86. The SDI values presented in this paper can be found at http://shiny.fzp.czu.cz/KVHEM/drought/.

## Electronic supplementary material

Supplementary Material
