# Supplementary material for: Revisiting the recent European droughts from a long-term perspective
Source: Sci Rep. 2018 Jun 22;8:9499. doi: 10.1038/s41598-018-27464-4 (PMC6015036; doi:10.1038/s41598-018-27464-4)
Supplement: Supplementary file 1 — Supplementary Material [file 41598_2018_27464_MOESM1_ESM.pdf]

Revisiting the recent European droughts from a long-term perspective

Martin Hanel[1,+,*], Oldřich Rakovec[2,1,+], Yannis Markonis[1,+], Petr Máca[1], Luis Samaniego[2], Jan Kyselý[1,3], and Rohini Kumar[2,+]

[1]Czech University of Life Sciences, Faculty of Environmental Sciences, Prague, 169 00, Czech Republic

[2]UFZ-Helmholtz Centre for Environmental Research, Leipzig, 04318, Germany

[3]Institute of Atmospheric Physics, Czech Academy of Sciences, Prague, 141 31, Czech Republic

[*]hanel@fzp.czu.cz

[+]These authors contributed equally to this work.

SUPPLEMENTARY MATERIAL

Contents

1 Evaluation of the model performance **2**

2 Effect of different potential evapotranspiration (PET) formulations on drought characteristics **5**

3 Trend significance **8**

4 Selected precipitation and soil moisture drought events **9**

5 The effect of time-varying threshold **12**

References **13**

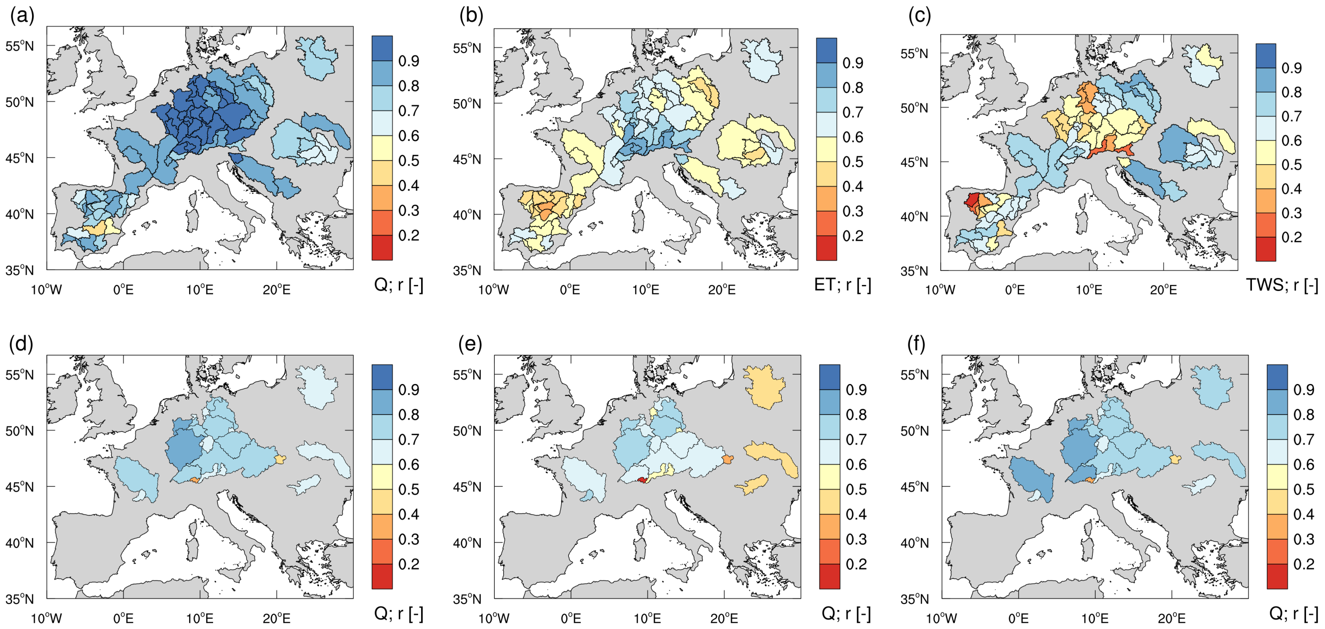

Figure S1. (top) Model performance in terms of correlation coefficients (r) for (a) routed streamflow (Q), (b) evapotranspiration (ET) and (c) terrestrial water storage (TWS) anomalies using monthly standardized values (83 basins presented by[1]) for the period 1950–2011. Note that the model is forced with E-OBS meteorological forcings. (bottom) Model performance in terms of correlation coefficients for monthly standardized routed streamflow covering the (d) entire, (e) pre-1900, and (f) post-1900 periods for the 29 basins with available long-term observations used in this study. Figure was created in NCL (ver. 6.2.1-1, https://www.ncl.ucar.edu/).

1 Evaluation of the model performance

The mHM setup follows the previous study of Rakovec et al.[1], in which the model parameterization is constrained against river discharge and the GRACE satellite-based terrestrial water storage (TWS)[1], and meteorological forcings are taken from the E-OBS datasets. The model exhibits a reasonable ability to capture the observed dynamics of monthly streamflow (Q), TWS and actual evapotranspiration (ET) during the available period of 1950–2011 (see Fig. S1; panels a, b, and c). The median skill in terms of correlations for standardized Q, TWS and ET estimated across 83 European river basins are 0.86, 0.62 and 0.60, respectively (Fig. S1; see[1] for more details). We would like to highlight that although we used correlation as an skill metric, it was estimated after standardising the variables of interest. This means that the seasonality component is removed and thus the predictive skill of the model variables can be considered free of its seasonal climate behaviour.

We also evaluate the model skill for discharge simulations at 29 gauging stations that have long observation records starting before 1900 (median lengths of 32 years before 1900 and 112 years after 1900) to gain more confidence in the modelling results regarding the historical reconstruction of water fluxes and states over the long period (1766–2015). In this respect, this step also provides an independent evaluation of the reconstructed precipitation and temperature fields[2,3]. The results of this analysis indicate a reasonably good model capability for capturing the observed dynamics of the standardized monthly discharge over the entire time period, as well as during the pre- and post-1900 periods (Fig. S1; panels d, e, and f). The median correlations

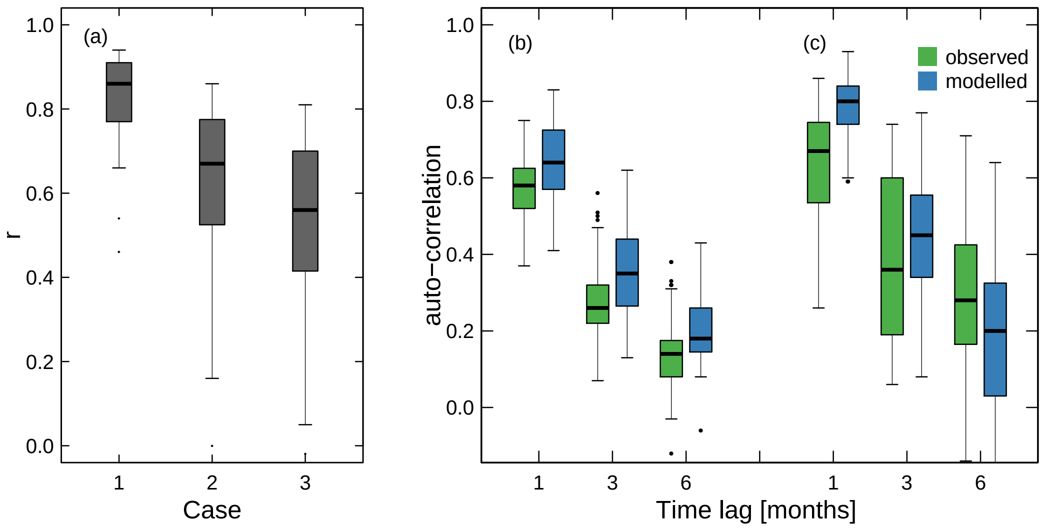

Figure S2. (a) Correlation coefficients for routed streamflow using monthly (Case 1) all standardized values (same as Figure S1, panel a), (Case 2) standardized values smaller than 0, and (Case 3) standardized values smaller than -0.5 across 83 basins for the period 1950–2011. Auto-correlation of standardized values for (b) observed and modelled routed streamflow (1950–2011) and (c) GRACE-based and modelled terrestrial water storage anomaly (2004–2011). All results are based on analysis/parameterization using mHM carried out by Rakovec et al.[1] using the E-OBS forcings. Figure was created in R (ver. 3.2.1, https://www.r-project.org/).

estimated across the 29 basins over these periods are 0.69 (1766–2015), 0.62 (1766–1899) and 0.74 (1900–2015). Note that even if the forcing data were perfectly correct, a difference in model performance would be expected due to physical changes in catchments. The decrease in model performance during the pre-1900 period can also be attributed to uncertainty in the reconstruction of precipitation and temperature fields[3], as well as to limited discharge data (especially in the eastern EU river basins), among other factors.

We recognize that our historical reconstruction of drought events based on the mHM simulations does not account for changes in land-cover scenes prior to 1990, as we use the fixed EU-wide 1990 CORINE (`http://www.eea.europa.eu`) land-cover dataset. It is worth noting that despite this limitation, the model skill for the discharge simulations across major gauging stations (with long records of more than 150 years) is quite reasonable (see Fig. S1; panels d, e, and f). By isolating the effects of other secondary variables such as land-cover/land-use changes, our aim here is to understand the impact of climate variability on resulting hydrological fluxes and states and their extreme behaviours, particularly drought events.

Furthermore, we evaluate the model performance of[1] with particular focus on low flow regimes. Therefore, Fig. S2a shows the correlation coefficients using monthly (Case 1) all standardized streamflow values (same as Fig. S1a), (Case 2) standardized values below a value 0 (dry anomalies), and (Case 3) standardized values below a threshold value -0.5. The last case usually represents a threshold for a start of drought condition (see U.S. Drought Monitor). It is obvious that the model performance slightly deteriorates towards drier anomalies conditions, however, there is still a considerable model skill for simulating the below normal conditions. Note that the inter-quartile range spans between 0.5 and 0.75 for the negative anomalies (Case 2) and between 0.4 and 0.7 for the even more drier conditions (Case 3).

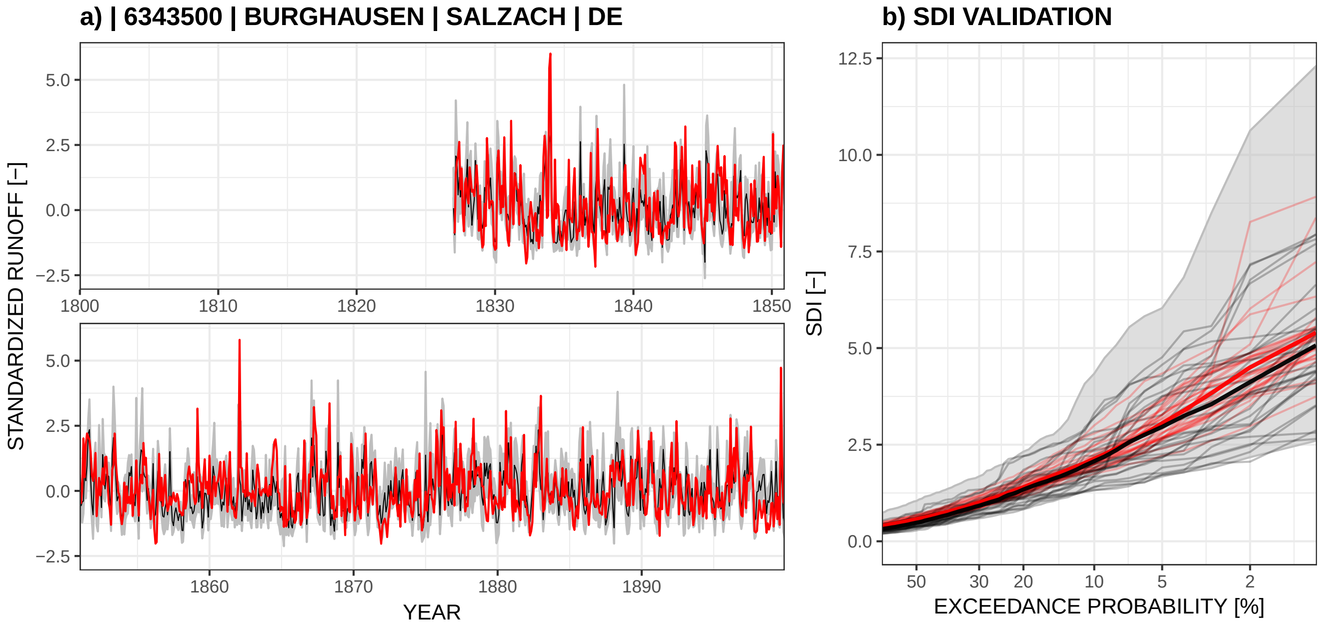

Figure S3. (a) Example time series of standardized routed runoff for the Salzach river at Burghausen (Germany). The red line corresponds to standardized observed runoff, and the black line to standardized ensemble average simulated routed runoff; the gray polygon indicates the 90% envelope for the mHM ensemble. (b) Distribution of the SDI for the observed (thin red lines) and mHM simulated (thin black lines) routed runoff. The thick lines correspond to the average over all considered catchments, and the grey area indicates the 90% envelope of the SDI distribution for all ensemble members. Figure was created in R (ver. 3.2.1, https://www.r-project.org/).

Fig. S2 panels b,c further show the model performance in terms of the auto-correlation at multiple time lags. This evaluation is considered to understand the recession behaviour of runoff hydrograph and TWS anomalies. Model exhibits slightly higher values than the observations, but overall a general tendency of decreasing correlation with increasing lag time is consistent between model and observations. Since we do not have consistent set of soil moisture observations, we use the terrestrial water storage anomaly observations from GRACE satellite to evaluate the skill of model to capture the auto-correlation behaviour. Note that the TWS has considerably lower sample size due to shorter data availability (monthly samples between 2002–2011). In this case also, the modelled estimates are on average higher compared to the observation – but overall the match between them are reasonably good. We also find a relatively larger spread in both modelled and observed auto-correlation estimates for the TWS anomalies as compared to those obtained for the streamflow values – which do reflect the basin to basin diversity in terms of TWS values. Example time-series of standardized observed and reconstructed routed runoff for the Salzach river at Burghausen (Germany) are given in Fig. S3a. Overall, the mHM simulation agrees well with observations. Fig. S3b compares the distribution of SDI derived from the mHM simulation to those from the observed runoff for the same 29 stations as in Fig. S1. The average and the spread of the simulated SDI values correspond well with the observed SDI values.

We show here also the validation results for the groundwater module of the mHM model (Fig. S4). Using the example from South Germany, we demonstrate that even the low complexity groundwater model is able to capture spatial mean of observed groundwater heads reasonably well. Note that the same holds also for capturing the patterns of regional variability (not shown).

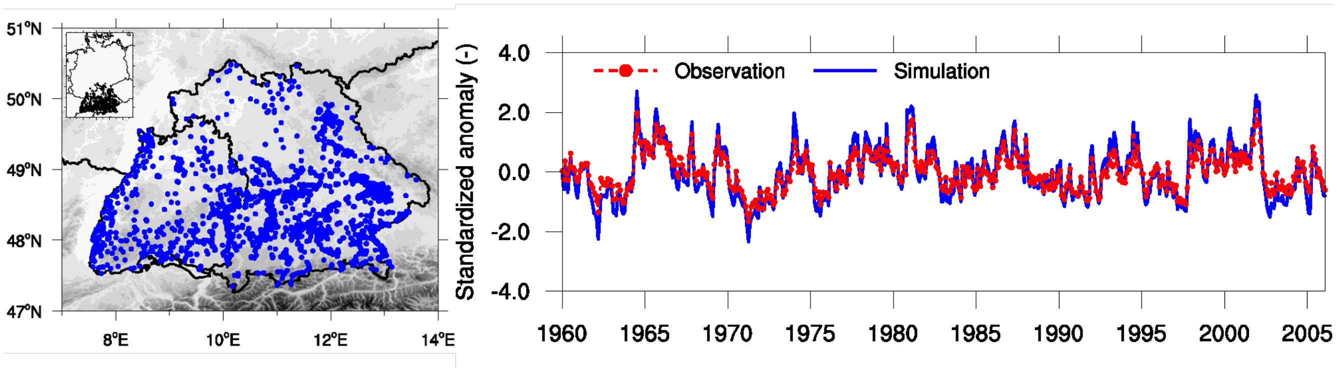

Figure S4. Evaluation of modelled standardized anomalies of groundwater levels against observations. (left) Location of groundwater wells (N=1991) located in South Germany with monthly time-series record ranging from 1960 to 2005. (right) Monthly dynamics of observed and simulated standardised anomalies of groundwater levels for assessing the skill of the model to capture the regional mean of observed groundwater levels. Figure was created in R (ver. 3.2.1, https://www.r-project.org/).

Further details on the groundwater drought study using this dataset can be found in our recent publications[4,5].

2 Effect of different potential evapotranspiration (PET) formulations on drought characteristics

Here, we briefly explore the effects of PET estimation on drought characteristics. For our 1766–2015 drought reconstruction, the PET is estimated from reconstructed monthly mean temperature fields[3] and potential extraterrestrial solar radiation[6] (denoted CASTY PET). For the period after 1900, the PET derived from half-degree gridded absolute values of mean, minimum and maximum temperature, vapour pressure and cloudiness and from a fixed monthly climatology for wind speed[7] is available in CRU TS[8] (denoted CRU PET). The evolutions of PET and precipitation minus PET are compared in Fig. S5; the effects on standardized grid cell runoff and soil moisture and their characteristics are demonstrated for Central Europe (Fig. S6) and the Mediterranean (Fig. S7). In all cases, the differences are small.

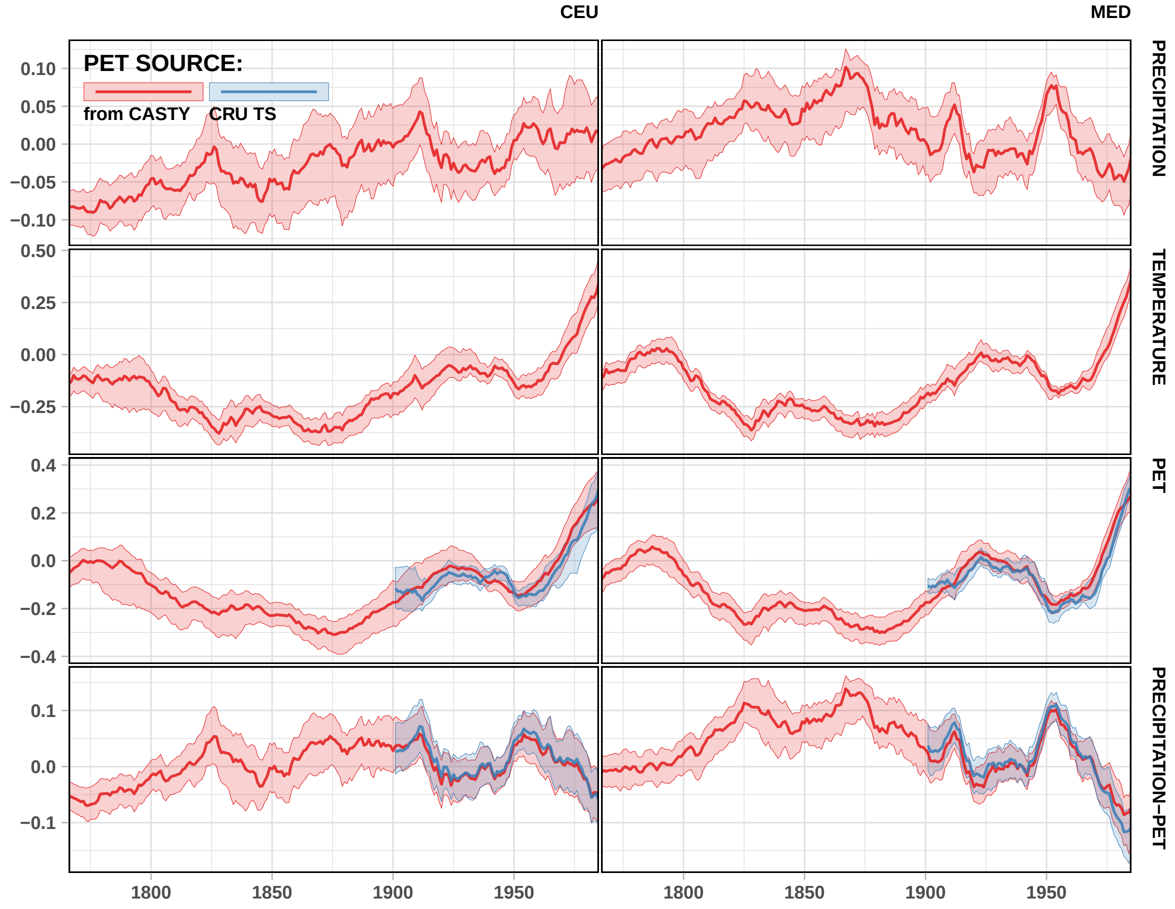

Figure S5. Temporal variation of 30-year moving average standardized precipitation, temperature, potential evapotranspiration (PET) and precipitation minus PET (in rows) for the Central Europe (CEU) and Mediterranean (MED) regions (in columns). The thick lines correspond to areal means, and the envelope spans the range between the 5th and 95th percentiles of grid cell values for each region. The CASTY PET is given in red and the CRU PET in blue colour. Figure was created in R (ver. 3.2.1, https://www.r-project.org/).

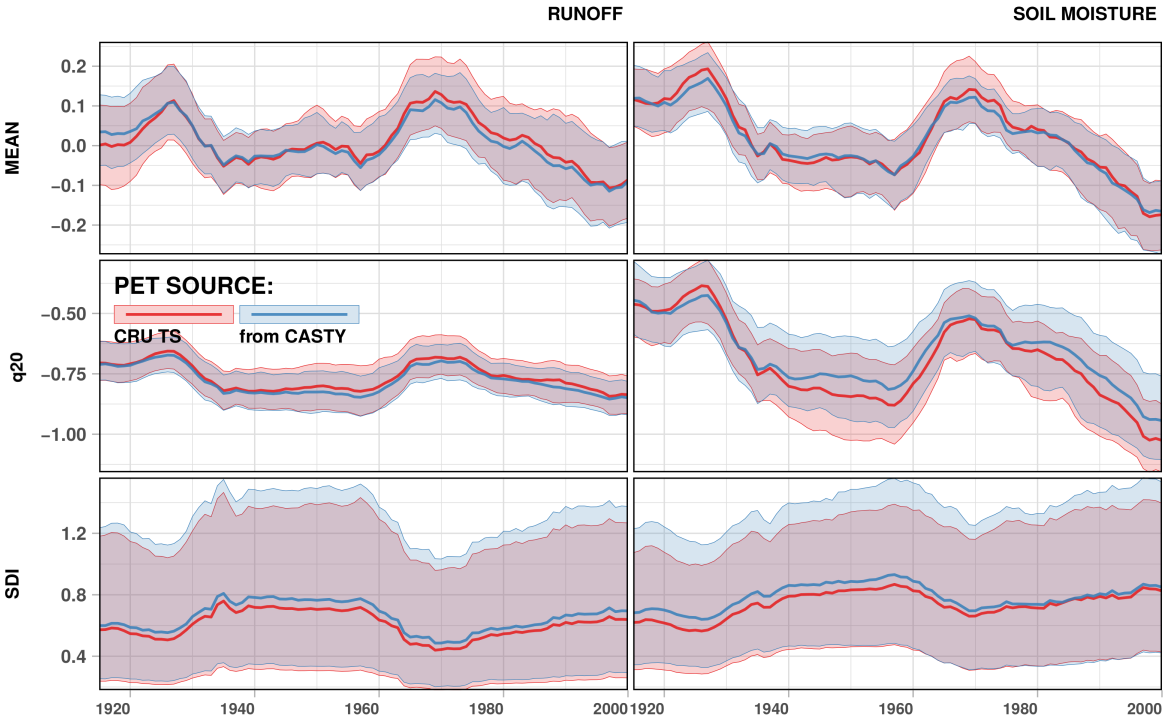

Figure S6. Comparison of the evolution of 30-year moving average mean (top), 20th percentile (middle) and standardized deficit index (SDI; bottom) for grid cell runoff (left) and soil moisture (right) for Central Europe (CEU). The results based on CASTY PET are shown in blue and CRU PET in red colour. Figure was created in R (ver. 3.2.1, https://www.r-project.org/).

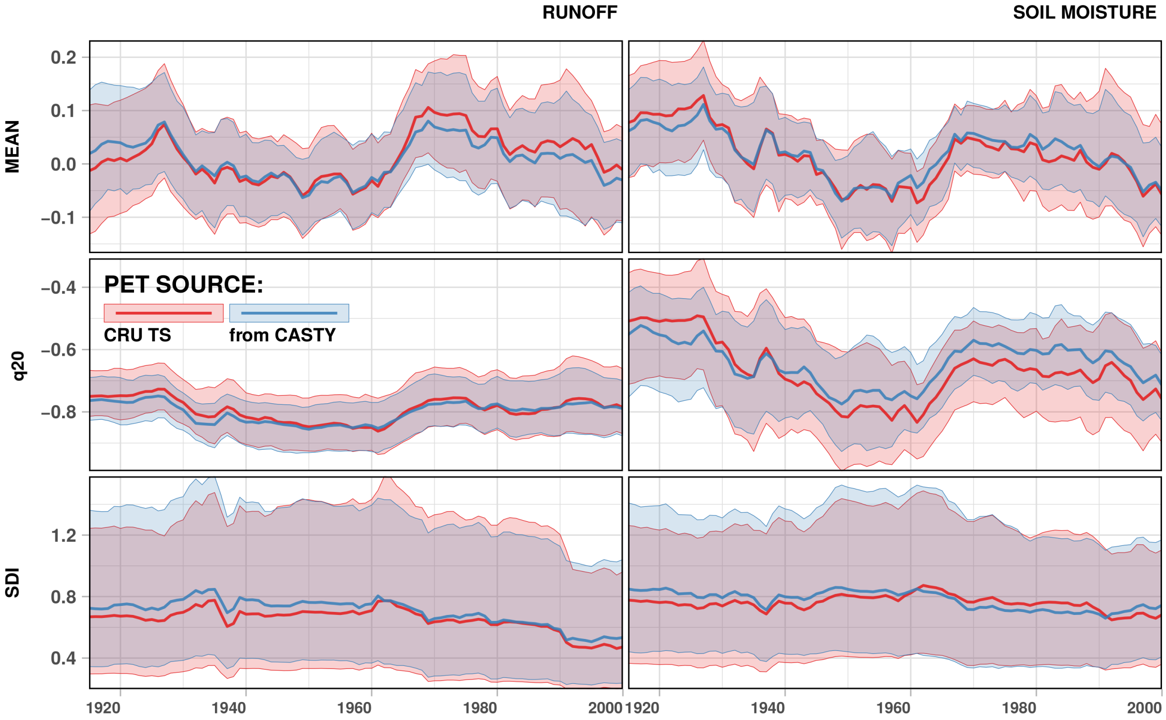

Figure S7. As Fig. S6 but for the Mediterranean (MED). Figure was created in R (ver. 3.2.1, https://www.r-project.org/).

3 Trend significance

We test the significance of trends in various characteristics of precipitation, soil moisture and runoff. The magnitude of the trend is determined by the Theil–Sen slope estimator, i.e., the median of the slope estimates obtained from all distinct pairs of points in the time series[9]. The significance of the trend is evaluated using a modification accounting for auto-correlation[10]. If not stated otherwise, the reported significance refers to the 0.1 significance level.

The significance of the trends in the average, 20th percentile (q_{20}) and SDI for standardized precipitation, grid cell runoff and soil moisture is tested at each grid cell. Table S1 reports the fraction of grid cells with significant increasing or decreasing trends over the CEU and MED regions. Fig. S8 shows the localization of the grid cells with significant trends together with their magnitudes.

The standardized precipitation anomalies over a relatively long time span of 250 years (1766–2015) slightly increase for the CEU and decrease for the MED regions. These trends are not statistically significant (at the 0.1 significance level) over most of the domain with respect to the whole period (see Fig. S8). However, there has been a pronounced reduction in precipitation in the MED region since 1970, which is consistent with the findings of previous observation-based studies[11,12]. The dynamics of the standardized grid cell runoff anomalies closely follow those of precipitation (the trend is not statistically significant as well), while a substantial drying trend is observed in soil moisture anomalies across both regions since the beginning of the 20th century. This trend is statistically significant for 95% of the MED area and 70% of the CEU area (Table S1).

Regardless of the increase in mean precipitation and grid cell runoff in CEU, the lower quantiles of the distribution are decreasing for all variables and regions. The strongest decreasing trend of the 20th percentile (q_{20}) is noticed in soil moisture, followed by precipitation, and the least in grid cell runoff, across both the CEU and MED regions. The trends are significant over more than 90% of the MED area for all variables and more than 30%, 60% and 75% of the CEU area for grid cell runoff, precipitation and soil moisture, respectively (Table S1).

In general, the fraction of grid cells with significant trends is larger for MED than CEU, and the trend magnitudes are larger in MED, especially for the case of a decrease in q_{20} and an increase in SDI.

Table S1. Fraction of grid cells with significant positive (↑) and negative (↓) trends in the average, 20th percentile (q_{20}) and SDI for precipitation (P), grid cell runoff (Q) and soil moisture (SM) over the period 1766–2015. Figure was created in R (ver. 3.2.1, https://www.r-project.org/).

		average↑	average↓	q_{20} ↑	q_{20} ↓	SDI↑	SDI↓
MED	precipitation	0.09	0.27	0.00	0.98	0.69	0.00
CEU		0.37	0.00	0.00	0.61	0.52	0.00
MED	grid cell runoff	0.18	0.08	0.00	0.91	0.43	0.00
CEU		0.33	0.01	0.04	0.32	0.11	0.00
MED	soil moisture	0.01	0.94	0.00	0.98	0.82	0.00
CEU		0.00	0.72	0.00	0.74	0.61	0.00

The substantial decrease in the soil moisture during the 20th century may be a reflection of global warming over this period (Fig. S5), which led to increased evaporative demand and reduced water availability in soil[13]. Within this context, we

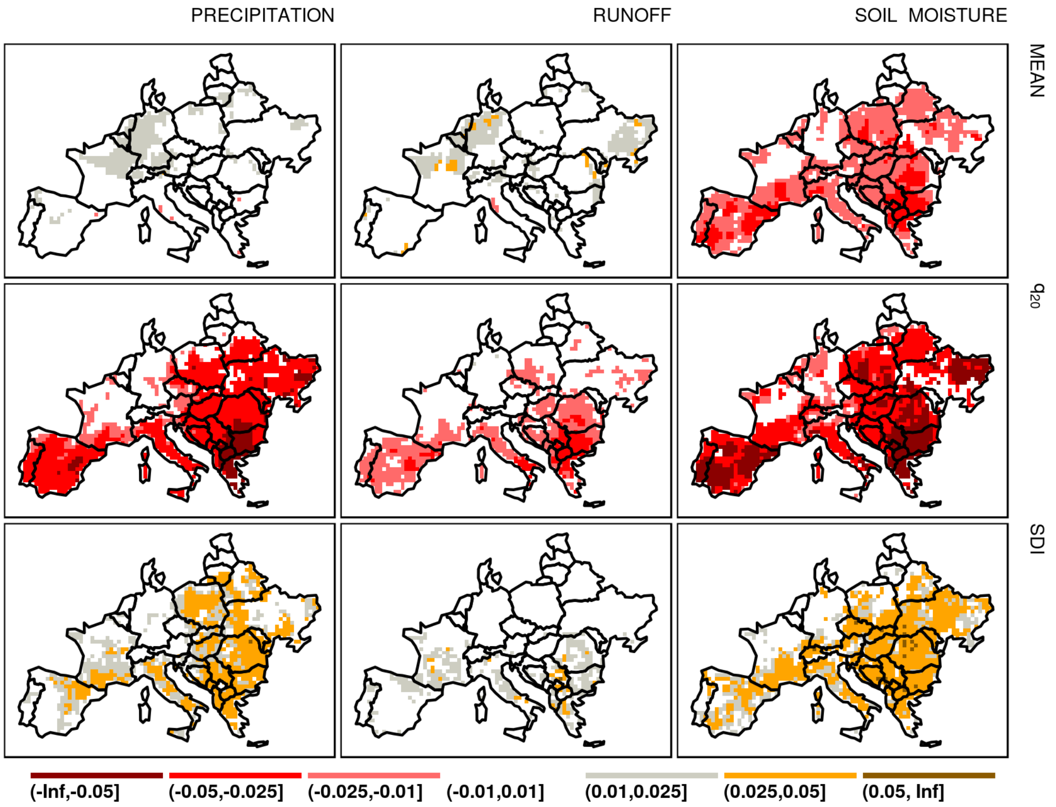

Figure S8. The magnitude of trends (standardized value per 10 years) significant at the 0.1 level. Figure was created in R (ver. 3.2.1, https://www.r-project.org/).

recognize the limitation of the temperature-based formulation of potential evapotranspiration (PET) as well as of the mHM in accounting for detailed vegetation feedback loops[14] that could have exaggerated this drying soil moisture trend to some degree[15]. The usage of more advanced PET estimation methods (such as Penman-Monteith) is not possible since the other atmospheric variables (e.g., short/long-wave radiation, wind speed, and relative humidity) are not yet available over 1766–2015. To assess the uncertainty due to differences in PET formulations, we perform additional simulations with a variant of the Penman-Monteith formulation from the CRU dataset (1901–2015). Despite differences in PET values for individual months and grid cells during the overlapping period (1901–2015), at longer time scales, only small differences are found in the temporal variability of standardized PET, precipitation deficit (precipitation minus PET), grid cell runoff and soil moisture anomalies (Fig. S5). The temperature-based PET consistently leads, however, to a slightly larger SDI (up to 6% for grid cell runoff and 3% for soil moisture; see Figs. S6-S7).

4 Selected precipitation and soil moisture drought events

Here, we present the exceedance probability maps for precipitation (Fig. S9) and soil moisture (Fig. S10) for the extreme drought events selected using the approach described in Methods Section in the main text, i.e., the events of 1822, 1858, 1874, 1921, 1943, 1947 and 1954 together with those of 2003 and 2015. Fig. S11 compares density estimates of SDI over the area for 1921, 2003 and 2015.

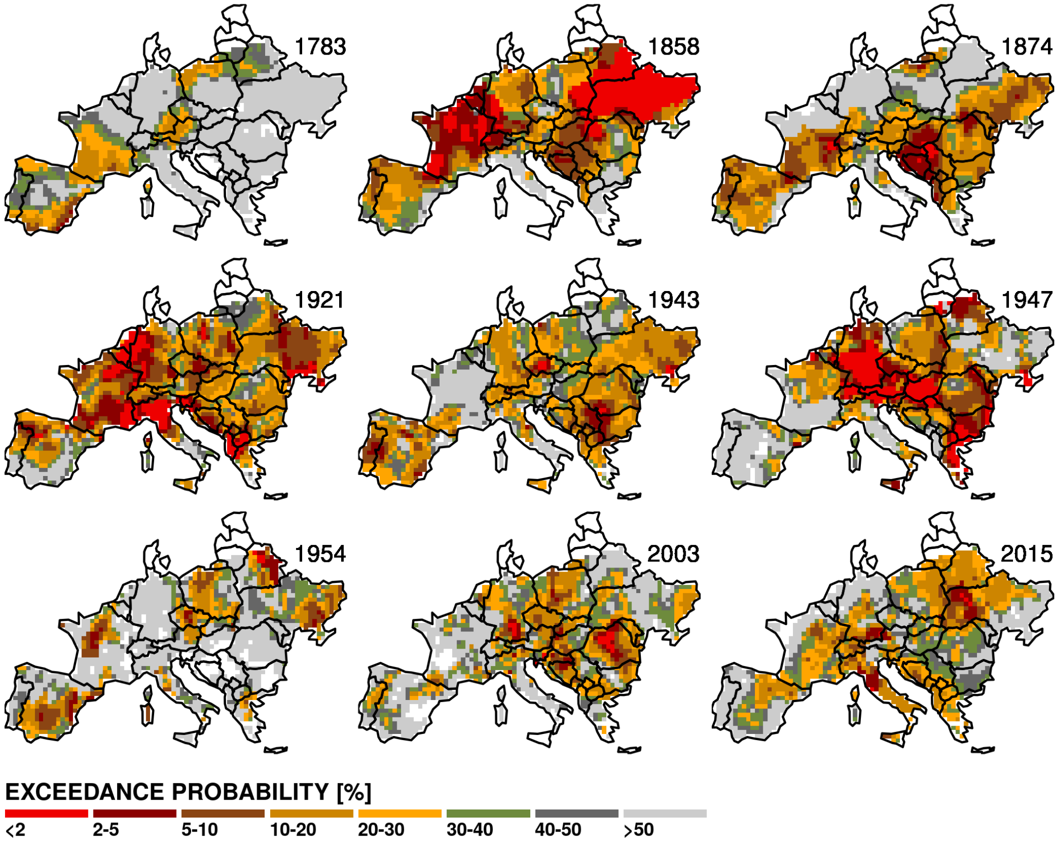

Figure S9. Exceedance probability of SDI for precipitation for selected extreme drought events together with those for 2003 and 2015. Note that the events are selected on the basis of grid cell runoff drought. Figure was created in R (ver. 3.2.1, https://www.r-project.org/).

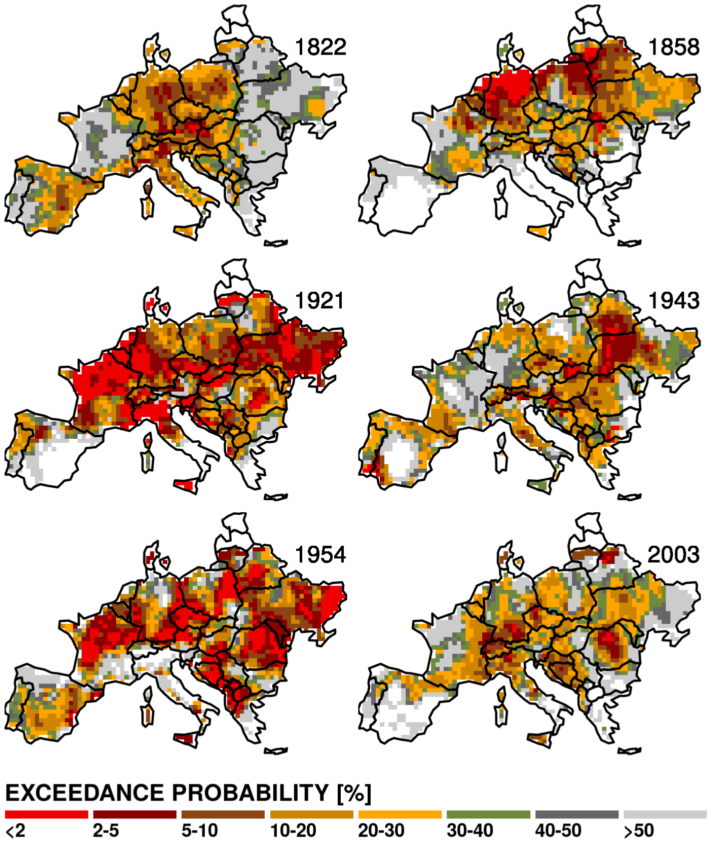

Figure S10. As in Fig. S9 but for soil moisture SDI. Figure was created in R (ver. 3.2.1, https://www.r-project.org/).

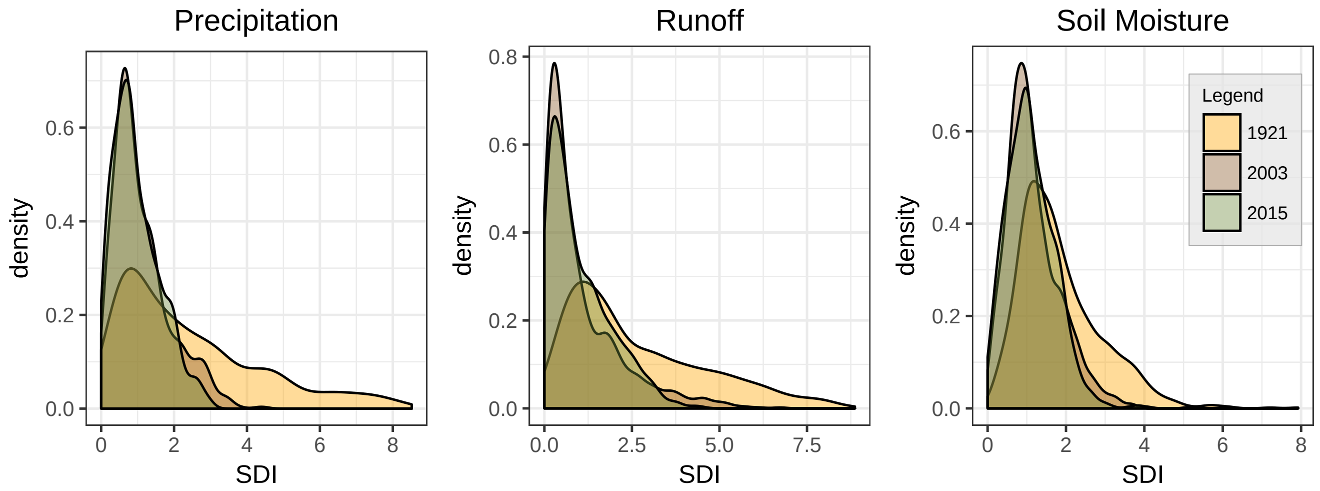

Figure S11. Comparison of the empirical distributions of the annual SDIs over grid cells for the years of 1921, 2003 and 2015. Kernel densities are used for the estimation of the distributions. Figure was created in R (ver. 3.2.1, https://www.r-project.org/).

5 The effect of time-varying threshold

We use a constant threshold (the 20th percentile – q_{20} – based on the entire 1766–2015 period) to define the SDI to assess the robustness of our results against the choice of the threshold. A constant threshold is frequently used when time series of standard lengths (30–50 years) are examined. In studies considering longer periods and non-stationary climate, a time-varying threshold is suggested[16,17].

Using the constant threshold, we recalculate the significance of trends in the SDI for each grid cell as in Supplementary Section 3. In general, the fraction of grid cells with significant increasing trends and the trend magnitudes are larger. This result is expected since a decrease in q_{20} in time (cf. Fig. 2 in the main text) means that the value of grid cell runoff is more often below the threshold towards the end of the series. On the other hand, the exceedance probabilities for the individual drought events (as shown in Fig. 5 and Supplementary Fig. S9 and Fig. S10) do not change much when the constant threshold is used. This is demonstrated in Fig. S12 comparing the exceedance probability for the 1921, 2003 and 2015 events for time-varying and constant thresholds.

Finally, the time-varying threshold reduces potential biases in the variability of reconstructed precipitation (and thus SDI) due to the low number of available stations with long records, which is the case in MED and eastern CEU regions[3], where the changes in the q_{20} are the most severe.

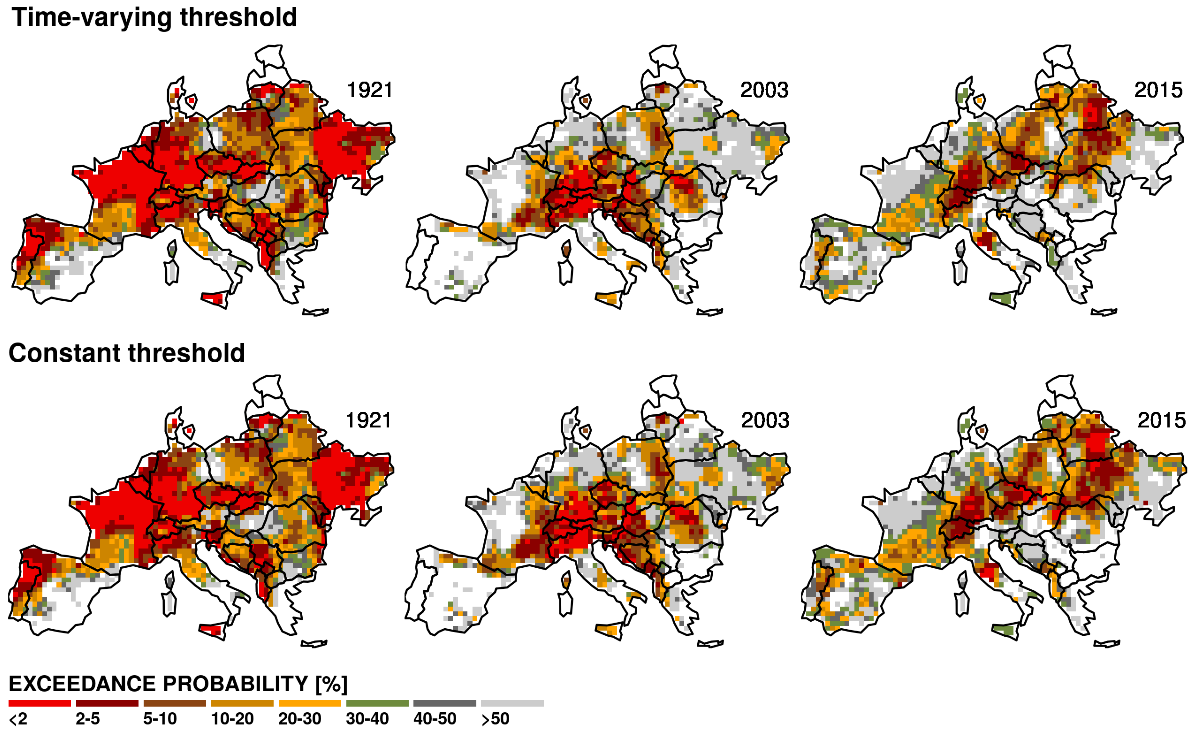

Figure S12. Exceedance probability of SDI for grid cell runoff based on time-varying (top row) and constant (bottom row) thresholds. Figure was created in R (ver. 3.2.1, https://www.r-project.org/).

References

1. Rakovec, O., Kumar, R., Attinger, S. & Samaniego, L. Improving the realism of hydrologic model functioning through multivariate parameter estimation. *Water Resour. Res.* (2016).
2. Casty, C., Handorf, D. & Sempf, M. Combined winter climate regimes over the North Atlantic/European sector 1766–2000. *Geophys. Res. Lett.* **32** (2005). L13801.
3. Casty, C., Raible, C. C., Stocker, T. F., Wanner, H. & Luterbacher, J. A European pattern climatology 1766–2000. *Clim. Dyn.* **29**, 791–805 (2007).
4. Kumar, R. *et al.* Multiscale evaluation of the standardized precipitation index as a groundwater drought indicator. *Hydrol. Earth Syst. Sci.* **20**, 1117 (2016).
5. Van Loon, A. F., Kumar, R. & Mishra, V. Testing the use of standardised indices and GRACE satellite data to estimate the European 2015 groundwater drought in near-real time. *Hydrol. Earth Syst. Sci.* **21**, 1947–1971 (2017).
6. Oudin, L. *et al.* Which potential evapotranspiration input for a lumped rainfall–runoff model?: Part 2—Towards a simple and efficient potential evapotranspiration model for rainfall–runoff modelling. *J. Hydrol.* **303**, 290–306 (2005).
7. New, M., Hulme, M. & Jones, P. Representing twentieth-century space–time climate variability. Part I: Development of a 1961–90 mean monthly terrestrial climatology. *J. Clim.* **12**, 829–856 (1999).
8. Harris, I., Jones, P., Osborn, T. & Lister, D. Updated high-resolution grids of monthly climatic observations – the CRU TS3.10 Dataset. *Int. J. Climatol.* **34**, 623–642 (2013).
9. Sen, P. K. Estimates of the Regression Coefficient Based on Kendall's Tau. *J. Am. Stat. Assoc.* **63**, 1379–1389 (1968).
10. Yue, S., Pilon, P., Phinney, B. & Cavadias, G. The influence of autocorrelation on the ability to detect trend in hydrological series. *Hydrol. Process.* **16**, 1807–1829 (2002).
11. Sousa, P. *et al.* Trends and extremes of drought indices throughout the 20th century in the Mediterranean. *Nat. Hazards Earth Syst. Sci.* **11**, 33–51 (2011).
12. Seneviratne, S. I. *et al.* *Changes in climate extremes and their impacts on the natural physical environment* (Cambridge University Press, 2012).
13. Dai, A., Trenberth, K. E. & Qian, T. A global dataset of Palmer Drought Severity Index for 1870–2002: relationship with soil moisture and effects of surface warming. *J. Hydrometeorol.* **5**, 1117–1130 (2004).
14. Prudhomme, C. *et al.* Hydrological droughts in the 21st century, hotspots and uncertainties from a global multimodel ensemble experiment. *Proc. Natl. Acad. Sci.* **111**, 3262–3267 (2014).
15. Sheffield, J., Wood, E. F. & Roderick, M. L. Little change in global drought over the past 60 years. *Nat.* **491**, 435–438 (2012).
16. Van Huijgevoort, M., Van Lanen, H., Teuling, A. & Uijlenhoet, R. Identification of changes in hydrological drought characteristics from a multi-GCM driven ensemble constrained by observed discharge. *J. Hydrol.* **512**, 421–434 (2014).
17. Wanders, N., Wada, Y. & Van Lanen, H. Global hydrological droughts in the 21st century under a changing hydrological regime. *Earth Syst. Dyn.* **6**, 1 (2015).
